# Investigating the (Mis)Match between Natural Pest Control Knowledge and the Intensity of Pesticide Use

**DOI:** 10.3390/insects9010002

**Published:** 2018-01-05

**Authors:** David Mall, Ashley E. Larsen, Emily A. Martin

**Affiliations:** 1 Department of Animal Ecology and Tropical Biology, Biocenter, University of Würzburg, Am Hubland, D-97074 Würzburg, Germany; david.mall@posteo.de; 2 Bren School of Environmental Science & Management, University of California, Santa Barbara, CA 93106-5131, USA; larsen@bren.ucsb.edu

**Keywords:** agroecology, agricultural intensity, biological pest control, crop, ecological intensification, insecticides, study system

## Abstract

Transforming modern agriculture towards both higher yields and greater sustainability is critical for preserving biodiversity in an increasingly populous and variable world. However, the intensity of agricultural practices varies strongly between crop systems. Given limited research capacity, it is crucial to focus efforts to increase sustainability in the crop systems that need it most. In this study, we investigate the match (or mismatch) between the intensity of pesticide use and the availability of knowledge on the ecosystem service of natural pest control across various crop systems. Using a systematic literature search on pest control and publicly available pesticide data, we find that pest control literature is not more abundant in crops where insecticide input per hectare is highest. Instead, pest control literature is most abundant, with the highest number of studies published, in crops with comparatively low insecticide input per hectare but with high world harvested area. These results suggest that a major increase of interest in agroecological research towards crops with high insecticide input, particularly cotton and horticultural crops such as citrus and high value-added vegetables, would help meet knowledge needs for a timely ecointensification of agriculture.

## 1. Introduction

Chemical pesticides are a cheap and efficient way for farmers to address pest problems. Yet, the social and environmental costs of pesticide use are increasingly evident in problems as far ranging as human birth outcomes, air pollution and pollinator declines [1]. Most importantly, chemical pesticides are not the only option to cope with different pests and to achieve high yields. Methods like crop rotation or sparing flowering or semi-natural areas in farmland have the potential to increase ecosystem services providing higher yields without pesticides [2,3,4]. This is particularly true of the ecosystem service of natural pest control. However, promoting natural pest control in practice is not straightforward, as appropriate measures vary strongly with region, farming system and crop and their long term success is currently uncertain [5,6,7].

Organic agriculture is hailed as one way to implement the integration of low-input agricultural systems into the natural environment. To date ca. one percent of worldwide agricultural land is under organic cultivation but this area has increased from 14.9 million hectares in 2000 to 50.9 million hectares in 2015 with positive outlook [8]. However, the ability of organic agriculture to meet the demand for world production in the next decades is under debate, one reason being that it is unclear whether organic yields can be comparable to conventional ones in many systems [9,10,11]. To accelerate the process of reducing chemical inputs beyond the organic sector and to limit mistakes in this fast-emerging field, scientific feedback and support are needed to fill crucial knowledge gaps.

On one hand, research on the mechanisms of biodiversity maintenance, ecosystem functioning and service provision across space and time provides the basis for long-term understanding and prediction under variable conditions [5,12,13]. On the other hand, applied research can help identify working strategies to alleviate negative impacts of intensive agriculture and harness the benefits of ecosystem services [2,4,14].

While such strategies are necessarily tailored to distinct regions due to variation in pest and enemy communities, production cycles and cultural norms, there are important crop-specific generalities in pesticide use. Crops vary in average pesticide use due to characteristics such as value, market (e.g., fresh or frozen) and pest load. As a result, forage crops such as alfalfa receive very low insecticide use, while high value fruit and nut crops can be treated with upwards of 40 kg/ha of insecticide product in a given year [15].

With such large variation in use, come similarly heterogeneous opportunities for ecological processes to provide substantial reductions in insecticide loads. In other words, the systems and crops ecologists choose or are hired to study determine in large part how effective agroecological insight will be for reducing agriculture inputs and reaping the on-farm and societal benefits of ecological intensification. In this paper, we use a systematic literature search and publicly available pesticide data to (1) highlight key crop and study systems where current understanding of natural pest control is comparatively low or lacking; (2) investigate to what extent crops selected for scientific study reflect the average intensity of crop cultivation practices. Due to limited global availability, data on amounts of pesticides applied in separate crops were restricted to Europe and the United States. We find that paradoxically, the number of recently published pest control studies was not highest in crops where pesticide use intensity is high, including particularly cotton and horticultural crops. Instead, studies focused on crops with globally important harvested area such as cereals, oil seeds and maize, where amounts of chemical agricultural inputs are comparatively low.

## 2. Materials and Methods

### 2.1. Systematic Literature Search

To obtain a systematic overview of available natural pest control literature, we performed a keyword search in the ISI Web of Science literature database. To ensure completeness, selected keywords where divided into four sections:

1. ‘agriculture’ as setting (as opposed to studies in natural systems)
2. ‘beneficial’ or ‘pest insects’ as focus of the study
3. factors potentially affecting natural pest control in crop fields, such as ‘landscape’ and ‘flower strips’
4. the systemic outcome of interest (‘pest control,’ ‘crop damage,’ ‘yield’).

Keywords in each section were then expanded to encompass a range of synonyms or related meanings, thus forming the complete search string (see Appendix).

The output contained 1739 articles covering a range from 1990 until 2016 (recorded 26 October 2017). Of these, 389 studies covering the most recent two publication years 2015/16 were included in further screening and analyses. Despite the small time frame represented, the studies published within the last two years account for 25% of the total studies on natural pest control since 2000 (1559 studies) and are most likely to inform on drivers and scales we now know to be important. The number of published articles per year in this search output has stayed relatively stable with <50 articles/year until 2006, after which it has increased sharply to triple this number (213) in 2016. The output was downloaded, read into Endnote and exported into an Excel sheet.

**Figure 1:**
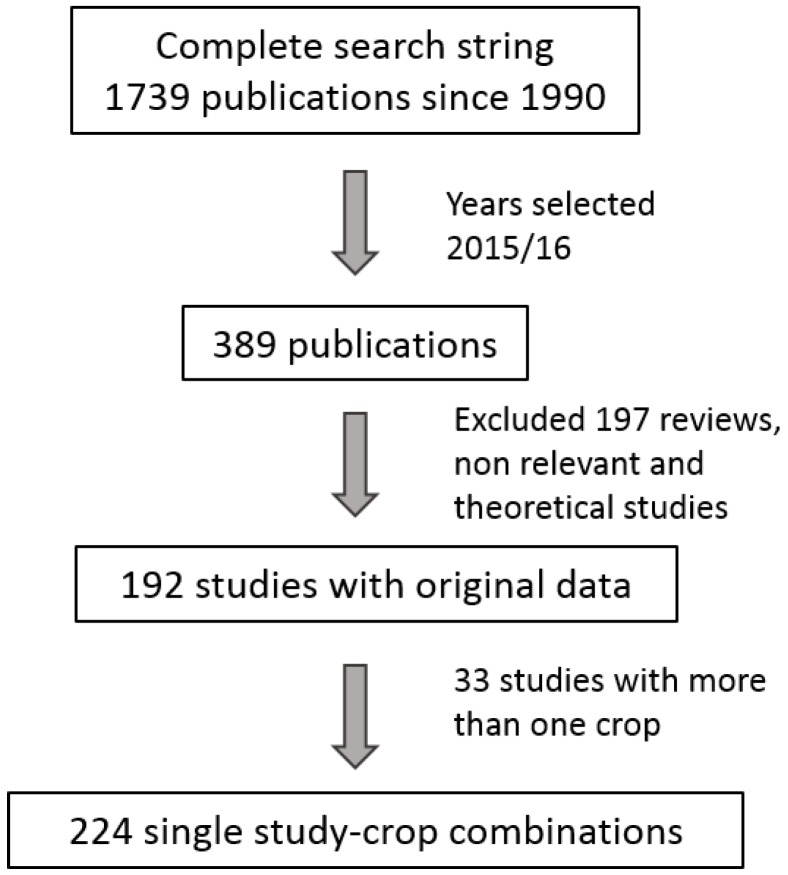
Selection procedure of the systematic literature search and resulting number of studies included.

We screened all 389 selected articles from 2015/16 manually for information not available in the search output. First, we classified each article into the categories study type, country and studied crop system. To be relevant, articles needed to contain sampling, experiments or observations on pest regulation mediated by non-chemical parameters like flower strips, intercropping, spraying refugia or landscape structure, or comparing effects of various levels of insecticide use such as organic or low-input vs. conventional or selective insecticides, with the exclusion of field toxicity trials (explanatory variables). Furthermore, response variables had to be connected to potential pest control indicators like densities of natural enemies, pests, crop damage or yield. Theoretical studies, studies restricted to the laboratory and reviews with no original data were not included. These criteria yielded 192 published articles (ca. 49%). Of these, 33 studies contained information for more than one crop. For those studies, information was used for each focal crop, resulting in 224 single study-crop combinations (Figure 1 and Table S1).

### 2.2. Pesticide Use Intensity Data

To compare literature data with the intensity of pesticide use in different cropping systems, we screened publicly available datasets for information on pesticide use per crop type. Official comprehensive and comparable data that distinguished pesticide use information at the crop level instead of aggregated statistics for all crops were surprisingly scarce and were unavailable at the global level. The most comprehensive comparable information was found for Europe and the USA in the form of (1) data provided to Eurostat by the European Crop Protection Agency (ECPA) [16] and (2) data of the United States Department of Agriculture (USDA) [17]. Both ECPA and USDA data are limited to a subset of globally or regionally important crops. Although information on other crops exists in some individual EU and non-EU countries, widely varying methodologies, years covered and units of measure make these difficult to combine in an overarching dataset. In a first step, we thus restricted information sources to the relatively homogeneous USDA and ECPA reports [16,17].

European data reported by the ECPA [16] are theoretical dosage estimates in kg of active ingredients (AI) per cultivated hectare, obtained by dividing estimated amounts of AI by the area under cultivation for each crop in the corresponding year. These data cover the years 1992–2003 and report pesticide usage (not sales) obtained using a combination of farmer panels, external and internal industry analyses performed for market purposes and submitted to the ECPA by product-selling companies. Data are provided for 12 crop categories including arable crops (cereals, maize, oilseeds, potatoes, sugar beet) and specialty crops (citrus, grapes, fruit trees, brassicas, cucurbits, tomatoes and other vegetable crops). Sulphur amounts are included in ECPA estimates as part of the fungicide category of pesticides.

Similarly, data reported by the USDA covering years 1960–2008 [17] are usage estimates in kg of AI per cultivated hectare and year (converted from pounds/acre). This report covers five crop categories in sufficient detail for our analysis (cereals, maize, soybean as oil seed, potatoes and cotton). Usage of insecticides, herbicides and fungicides (excluding sulphur and mineral oils) are reported.

In a second step, we sought to complete the dataset with further crops, particularly those for which information was available in Europe but not the USA. The database resource of the USDA National Agricultural Statistics Service [18] provided data for seven further crop groups. However, incomplete yearly coverage, very strong interannual variation in pesticide amounts in some crops and a vague description of how many and which states were included per data point led us to reject this database as an information source in this analysis. Instead, we obtained information on further crop groups in the USA from California Department of Pesticide Regulation Pesticide Use Reports (PUR) [19]. We used these data to estimate pesticide use for 10 additional crop groups, mirroring the available EU data. This California specific database is widely considered the most detailed and comprehensive pesticide use database available in the US [20]. Since California is one of 2–3 major US producers of these additional crops, values are likely to reflect input intensity at the country level and were thus used for comparison with European crops.

The California PUR data are individual records of actual agricultural pesticide use mandated by California law, covering the years 1990–2015. Sulphur, which accounts for over 20% of pesticide active ingredients by weight in California [19] and functions as both an insecticide and fungicide, was excluded from estimates of insecticides by excluding combined insecticides-fungicides from calculations. In contrast to ECPA and USDA sources, Californian data were calculated as kg of AI per harvested area, as planted areas are not available for all years and crops. Annual harvested area by crop group was aggregated from individual crop-level data from the County Agricultural Commissioner’s Report from 1990 to 2015. Differences between harvested (bearing) area and estimated total area (bearing + non-bearing, planted) from the USDA California Agricultural Statistics Survey for selected crop groups (grapes, citrus, rice and nuts) and years averaged less than 10% and did not qualitatively change patterns of pesticide use. Despite attempting to homogenize methodologies, estimated values on Californian crops were over 10-fold the values found in corresponding European crops, likely due in large part to differences in underlying chemicals in use and reporting requirements. We thus included these data for qualitative comparison but not in averaged estimates of insecticide use across regions (see below). Compiled pesticide estimates from all data sources are presented in Table S2.

The year 2003 showed the most recent comparable overlap between USDA and ECPA pesticide use data. As such, 2003 was used for comparison with the literature on natural pest control. To enable an overall comparison with the literature across regions, we pooled US and EU datasets by averaging values of the five crops reported by the USDA with corresponding crops of the ECPA dataset. Values for remaining crops were extracted from the ECPA dataset (Figure S1). As no information was available for oil seeds in 2003 in the USDA dataset, we used the value of 2004 for this crop type.

We approximated pesticide use intensity as the total input of AI per crop and year in kg/cultivated ha. Available data did not include pesticide application frequency at the crop level, which would allow more accurate representation of actual usage intensity (area-treatment estimates, [21]). However, total averaged measures have been shown to relate linearly to treated ha [22] and are likely to reflect the broad differences between crops in application intensity per ha. Pesticides for which information was available included insecticides, herbicides and fungicides. We restricted comparisons with the literature to only insecticide data because of their direct relevance to biocontrol and the preponderance of herbivore-focused agroecological studies. However, time trends for all pesticides are shown in order to place data within the context of the whole range of pesticide interventions used in each crop over time.

Total crop production and/or efficiency metrics (yields) were considered other potential drivers of agroecological research intensity. Additional data on global, European and US-level yield (kg/ha), production (tonnes), net production value (International $) and harvested area (ha) were thus compiled as covariates for all relevant crop aggregates from online resources of the Food and Agriculture Organization (FAO) [23]. These were selected for 2014 as the available year most closely corresponding the period covered by the literature search. FAO metrics for cotton (world harvested area and yield) were unavailable and we completed these using 2013/2014 data from the USDA foreign agricultural service cotton database [24]. Detailed methods and compiled production metrics are shown in Table S3.

### 2.3. Statistical Analyses

Although insecticide estimates were restricted to Europe and the USA, we considered the number of pest control studies performed in each crop to be relevant at the global level. Both the number of published pest control studies and FAO crop production data were strongly positively correlated between world-level and pooled EU and USA datasets (Pearson’s *r* >0.89, *p* < 0.001 for all but net production value; net production value *r* = 0.61, *p* = 0.03). We thus included world-level data in comparisons of these factors with insecticide use intensity.

The number of published pest control studies per crop aggregate was analysed as a function of insecticide use intensity and crop production metrics using a negative binomial generalized linear model with log link, accounting for overdispersion of the data. Crop production metrics were strongly collinear (Figure S2). In particular, harvested area and net production value were positively related to production and yields, respectively and led to variance inflation factors >3 when included in models. Thus, only yields (t/ha) and total production (kt) were included as covariates with insecticide intensity (kg/ha) (model 1). To illustrate the effect of correlated variables, we also applied a second model including only total harvested area of each crop (million ha in 2014) (model 2). The crop category ‘other vegetables’ as defined by the ECPA source represented an outlier with very high insecticide use intensity, which was not matched by high values in Californian data and for which a full list of detailed crops was unavailable for further comparison. We thus present results of model 1 excluding ‘other vegetables’; inclusion of this category leads to a non-significant negative relationship between insecticide use and the number of studies (estimates [95% CI] of effects of insecticide use, world yield and world production on number of studies, respectively: −0.13 [−0.37, 0.09], *p* = 0.28; −0.03 [−0.05, −0.01], *p* < 0.001; 0.47 × 10^−6^ [−0.15 × 10^−6^, 1.1 × 10^−6^], *p* = 0.14).

While insecticide data were only available for large-scale analysis until 2003, more recent changes in usage intensity may impact the number of studies published in later years. In order to examine the effect of changes in insecticide use post-2003, we calculated relative change in insecticide use from 2003 to 2015 for crops of the Californian dataset (the only dataset with information more recent than 2008). We used the % change calculated for Californian crops to impute estimated insecticide use in 2015 following: Insecticide_2015_ = Insecticide_2003_ * (1 + % change_2003−2015_/100). We reran analyses (model 1) using estimated values of insecticide use for 2015. Recent reductions in use detected in Californian crops since 2003 led to a non-significant negative relationship between the number of studies published and insecticide use intensity (estimates [95% CI] of effects of estimated insecticide use in 2015: −0.27 [−0.8,0.26], *p* = 0.31).

Models were checked graphically for homoscedasticity. All analyses were performed in R statistical software v. 3.4.1 [25].

## 3. Results

### 3.1. Systematic Literature Search

**Figure 2:**
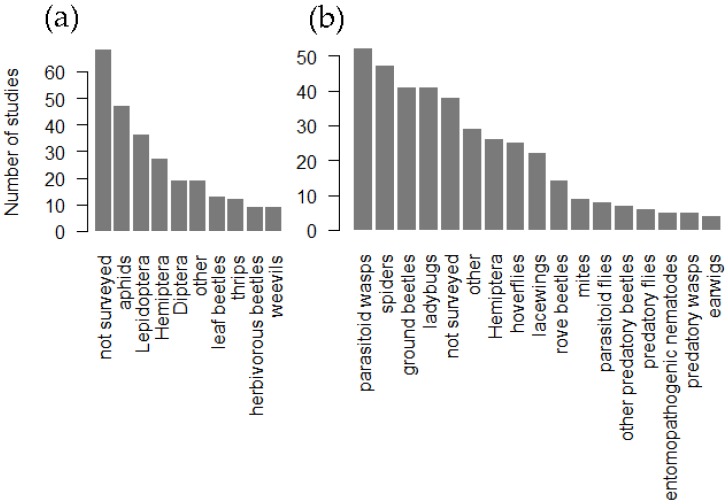
Distribution of (**a**) broad pest groups and (**b**) natural enemy groups examined by pest control-related studies in 2015/16. Hemiptera refers to all families except aphids (Hemiptera: Aphididae).

Overall, the 192 publications covered 39 crop systems in 41 countries (Figure S1 and Table S1). Of these, 36% did not examine pest groups but focused on other response variables, particularly natural enemies. Studied pests were dominated by aphids (47 studies), followed by pest larvae of Lepidoptera (36 studies) and strongly varied other Hemiptera (27 studies; groups include leaf and plant hoppers, stink, mirid, milkweed and shield bugs, scale insects and whiteflies) (Figure 2). Parasitoid wasps, spiders and ground beetles (Carabidae) followed by ladybugs (Coccinellidae) were the main focus of natural enemy studies.

The responses investigated by (broadly termed) pest control studies ranged from pest density and natural enemy observations to experimental measures of pest control provision using exclosures, predation cards or parasitism counts, to crop damage and yields (Table 1). Of these, 49% provided simultaneous measurement of pest and enemy populations or of crop damage and enemies. However, only 14% and 2% combined these with measures of pest control and yields, respectively, while five studies considered the additional ecosystem service of pollination.

Pest control studies on the three major crops, wheat (24 studies), maize (16) and soybean (13, included in oil seed plants (21)), together with fruit trees (17) take the top position in numbers published in 2015/16 (Figure S1). In addition, 65% of all studies were implemented in either Europe (70) or the USA (55).

### 3.2. Pesticide Use Intensity

**Figure 3:**
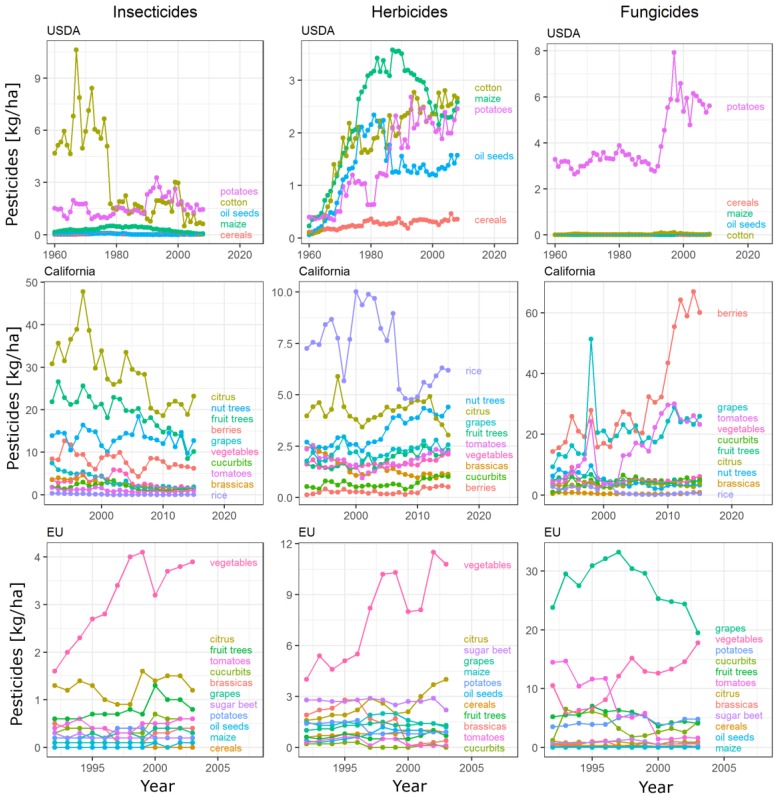
Pesticide amounts per crop type in kg of active ingredients/ha. Values are based on USDA, California PUR and ECPA sources from 1960 to 2008 (US data [17], **top panel**), 1990–2015 (California data [19], **middle panel**) and 1992–2003 (European data [16], **bottom panel**), respectively. Note varying axis scales for visibility. EU fungicide data include sulphur amounts but US data exclude them. To ease interpretation of overlapping time series, the order of legend crop names (top to bottom) corresponds to the order of last points (highest to lowest values) in the time series of each crop.

Pesticide use intensity as reported by ECPA (Europe), USDA (USA) and California sources varied considerably between crop groups and over time (Figure 3). Insecticide levels since 2000 were highest on various vegetable crops such as lettuce and carrots in the EU and citrus, nut and fruit trees in California. Excepting fruit trees, herbicide values were also highest for these crops as well as for rice in California. Similarly, the highest amounts of fungicides were found on potatoes in the US and vegetables, grapes and berries in California and the EU. Despite decreasing trends for insecticide on cotton (since 1980) and for fungicides on grapes (since 1998), overall trends show strong increases until 2003 in all three input types for field vegetables in the EU and in herbicides for three major crops (cotton, corn and potatoes) in the US, though insecticides appear to be decreasing in several crops in subsequent years in California (see Section 3.3). Amounts of input are relatively stable over time in other world crops but recent years show an apparent increase in herbicides in European citrus crops.

### 3.3. Relating Pest Control Studies to Pesticide Use Intensity in World Crops

**Figure 4:**
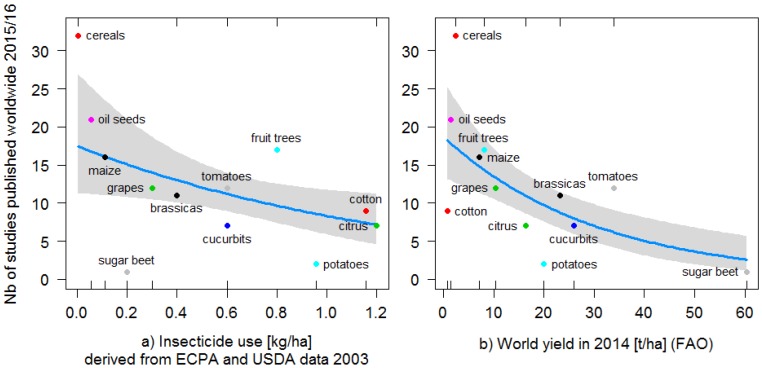
The number of studies on pest control detected in 2015/16 as a function of (**a**) insecticide usage in kg/ha derived from ECPA and USDA data from 2003; (**b**) the most recent yield data from the FAO in kg/ha from 2014. Predicted values of the negative binomial generalized linear model are shown (Model 1, blue lines; 95% confidence intervals are shown in grey). The category ‘other vegetables’ at 3.9 kg/ha of insecticides in 2003 (10 pest control studies published; yield estimated at 15.9 t/ha in 2014) was excluded from the model as an outlier for insecticides (see Methods).

The number of studies examining pest or pest control variables in separate crops was significantly negatively related to insecticide use intensity in these crops (Table 2 and Figure 4a). When recent reductions in insecticide use in Californian crops were accounted for and when the outlier category ‘other vegetables’ was included, the number of studies showed no relationship with insecticide use intensity (Methods; estimates [95% CI] of effects of estimated insecticide use in 2015: −0.27 [−0.8, 0.26], *p* = 0.31; effects including ‘other vegetables’: −0.13 [−0.37, 0.09], *p* = 0.28). Studies were also significantly less numerous in high-yielding crops (Figure 4b). In contrast, the number of pest control-related studies was highest when the harvested area of crops was high, such as in cereal, oilseed crops and maize (Figure S3).

## 4. Discussion

This study finds that despite strong interest in pest control research in recent years, agroecological knowledge gain has not focused on crops for which estimated insecticide (and overall pesticide) use intensity is high. Instead, research has been most intensive on crops such as cereals, oil seeds and maize that represent the bulk of cultivated agricultural surfaces [23], as well as the most important cash crops for a majority of farmers and agricultural marketing chains. Part of this finding may be due to the constraints of government and applied scientists (e.g., extension agents) directed to work on grain crops of importance to regional and global food security, rather than horticultural crops. However, if a goal beyond food security is to reduce local environmental harm stemming from intensive pesticide use, we suggest additional focus on high pesticide use crops is merited.

The preponderance of research focusing on high production, low use crops confirms the extensive literature approach of Thorn et al. [26] who found wheat, maize and rice to be the most studied crops worldwide in ecosystem service research. Focusing research on widespread, globally important crops has clear benefits for several reasons. First, such insights can be applied over vast spatial scales. Second, providing ecological solutions to pest problems in these crops can critically help to stabilize global production, agricultural prices and food security under changing environmental conditions. Third, research is facilitated by the widespread availability of these crops for study, particularly when performed at landscape to regional spatial scales. Importantly, the significance of these crops in terms of area covered means that they drive major world patterns of landscape simplification and habitat loss and are thus critical systems to investigate options for combining conservation of biodiversity and its functions with large-scale agricultural production [2,27].

However, we highlight that this focus on major cash crops limits our ability to improve production practices in less widespread crops. While in cereals, oil seeds and maize, insecticide use intensity is below 0.5 kg/ha in the USA since the 1960s, insecticide use in high intensity crops such as fresh vegetables, cotton, fruit and citrus trees, is both more variable and orders of magnitude higher (Figure 3). Concerns about environmental pollution and human health impacts of pesticides are most strongly associated with a high concentration of contaminants [1,28,29]. Thus, reducing insecticides by 10% on wheat, may have benefits to wheat farmers and reduce prices for consumers but the environmental and health benefits to society are likely higher from reducing extremely high levels of pesticide use on fruits and vegetables by 10%, even if doing so leads to less global reduction in total pesticide use. In other words, these crops represent ca. 120 million ha of harvested area worldwide [23] in which the cost of pesticide use for human health, biodiversity and ecosystem resilience to environmental change is likely to be disproportionately high.

While considerable progress is being made with notable reductions of insecticide use in recent years in horticultural crops such as fruit trees through the development of integrated production systems [30,31], many crops remain contingent on the use of high dosages or concentrations of selected active ingredients, for which licenses are costly and pest resistance is a concern [31]. In part, this dependence is due to global markets and generally assumed consumer preferences which make high intensity crops particularly vulnerable to damage by pests and diseases, even when such damage is cosmetic and has no or little impact on production [32,33]. Low damage tolerance combined with the agroeconomic efficacy of insecticides compared to alternative means of control make it difficult to devise practical solutions for producers. Solving this dilemma hinges on tailoring research to the biological, agronomic and economic challenges associated with these systems. We thus argue that in parallel with efforts to adapt consumption patterns to sustainably grown food, agroecological research needs to play a role in providing alternative means of production in crops with high insecticide input, in addition to major market crops where insecticide inputs are low.

The pest and enemy groups studied by the reviewed literature reflect this bias. Aphids and leaf beetles comprise main pest groups of cereal crops and together they were the focus of 32% of reviewed studies, with correspondingly high numbers focused on their main enemy groups (parasitoid wasps, spiders, predatory beetles including ladybugs). Although aphids can cause serious yield losses in a majority of cereal crops, considerable progress has been made in recent years to predict and control populations by means other than synthetic pesticide spraying (e.g., [33,34]). While continued research will further improve these methods, improvement of agricultural practices now depends greatly on knowledge uptake and implementation by practitioners [33]. In contrast, other crop-pest-enemy complexes studied by the ecological literature are highly diverse and include pests causing major yield losses in a large variety of crops.

Major variables considered in recent studies were the effects of landscape and local (field)-scale land use intensity on pests and natural enemies, as well as studies on climate [35]. However, as noted in previous reviews [36], only a minority of studies measured the full range of impact of these variables from their effects on pests and enemies, to pest control, crop damage and yields. These measures are critical to understand and optimize pest control provision and to increase adoption of natural pest control-friendly management practices in light of their direct benefits for crop yield [37,38].

We found both biocontrol research and available data on pesticide use per crop to be strongly biased towards Europe and the USA. This is in line with results of Steward et al. [39], who warn against neglecting to provide ecological solutions in areas that are both economically unstable and hotspots of biodiversity. The almost complete lack of reliable data on the intensity of pesticide use in separate crops in most tropical regions compounds the problem in this study by preventing an assessment of highest intensity crops, which limited research resources may focus on. However, total pesticide usage for all crops combined is estimated at ca. 2 to 15 times higher in South America and Asia than Europe and the USA [40], with extreme associated environmental costs. In combination with direct costs to human health [28,29], this makes research on and adoption of ecological intensification practices in these regions an ever more crucial endeavour.

The timeline of pesticide use for major US crops reflects the increasing uptake of genetically modified (GMO) crops for soybeans, corn and cotton. GMO’s were introduced in the late 1990s. GMO crops are genetically engineered for herbicide tolerance (‘round-up ready’ i.e., glyphosate-tolerant) or insect resistance (*Bacillus thuringiensis* [Bt] crops) and often have stacked genes for both [41]. By 2014, over 90% of soybeans, maize and cotton acres were planted in biotech varieties. Adopters of herbicide-resistant soybeans are estimated to use roughly 30% more herbicides than conventional soybeans, while adopters of GMO maize are only estimated to use roughly 1% more herbicides and 11% less insecticides than their conventional counterparts in the US [42]. These changes are illustrated by the time trends showing a decrease in insecticides for US maize and increase in herbicides for maize and oilseeds after the introduction of GMOs. However, differences between EU and US due to different levels of GMO adoption are not yet observable in 2003, the last year of overlapping data between the two regions.

Pesticides vary dramatically in their toxicity for different non-target outcomes [43] from acute and chronic human health to water quality and pollinator concerns. Here we use kg of active ingredients to identify crops with high use, as identifying which crops use insecticides that are the most environmentally damaging to outcomes of concern was not possible with widely available pesticide data. While high use of even low toxicity chemicals is likely to have a range of environmental externalities, understanding which crops use insecticides of particular toxicity would further enable ecologists to target ecological pest control strategies towards crops and pests with the greatest threat to ecosystem or human health.

## 5. Conclusions

Applying the most recent comparable insecticide data and our systematic literature search, the results point to a surprising mismatch between agroecological research effort and insecticide use intensity in different crops. Instead of increasing with insecticide use intensity, research efforts have focused on crops with high globally harvested area. Increasing knowledge in high insecticide use crops is fundamental to capturing the societal benefits of ecological intensification. Further, many of the most concerning environmental and human health costs due to pesticide use are suggested to occur in developing countries that may lack strong institutions and worker protections. However, understanding both agrochemical use and crop systems is hampered by a lack of pesticide use data and biological pest control research outside Europe and North America. Overall, the described situation urges towards further research intensification on high insecticide use crops and crop systems outside of the US and EU in order to catalyse fundamental improvements in the sustainability of agricultural systems worldwide.

## Figures and Tables

**Table 1:** Distribution of response variables examined by pest control-related studies in 2015/16. Letters refer to studies examining combinations of multiple response variables (see footnote).

Surveyed Response	Number of Studies
Enemies	152
Pests	122
Pest control	49
Crop damage	14
Yield	12
Pollinators/Pollination	5
NP ^1^	95
NPX ^1^	27
NPXY ^1^	4
Total	192

^1^ N: natural enemies; P: pests or crop damage; X: pest control; Y: yields.

**Table 2:** Results of negative binomial generalized linear models predicting the number of pest control studies in 2015/16 as a function of insecticide use intensity and world production metrics per crop. Significant terms are in bold. *n* = 12 (model 1, ‘other vegetables’ excluded; see Methods) and 13 (model 2) separate crop types, respectively. The combined effects of insecticide use, world yield and world production (model 1; Figure 4) and the converse effects of world harvested area only (model 2; Figure S3) are shown. Terms were examined in separate models due to correlation between harvested area and other metrics (Methods and Figure S2).

	Deviance	Residual Deviance	Estimate	Confidence Interval	*p*-Value
Model 1		49.7			
(Intercept)			3.31	2.64–3.97	**<0.001**
Insecticide use (kg/ha)	13.9	35.75	−0.74	−1.4–0.1	**0.022**
World yield 2014 (t/ha)	20.6	15.15	−0.03	−0.05–0.02	**<0.001**
World production 2014 (kt)	0	15.15	−1 × 10^−9^	−0.71 × 10^−6^−0.73 × 10^−6^	0.986
Model 2		26.86			
(Intercept)			1.5	0.88–2.14	**<0.001**
log(World harvested area 2014 (million ha))	11.1	15.8	0.28	0.11–0.45	**0.002**

## Supplementary Materials

The following are available online at www.mdpi.com/2075-4450/9/1/2/s1, Figure S1: Total number of pest control studies per crop, Figure S2: Pearson correlation matrix with all potential covariates, Figure S3: The number of studies on pest control as a function of total harvested area, Table S1: List of pest control-related studies obtained by systematic literature search for 2015/16, Table S2: Amounts of pesticide active ingredients used per hectare in crops in Europe and the USA. Table S3: FAO search methods and production metrics for crop types considered in analyses.

## Appendix

*Supplementary methods—Systematic literature search.* For each broad section of the systematic literature search, keywords were chosen based on consideration of importance and specificity. Synonyms and alternatives where included. The topic search (ts) was used. The keyword sections were linked with the Boolean AND operator and the keywords within the sections by OR. Truncation (*) was only used when including plural or slightly different word endings made sense. Quotation marks were used to search for exact word combinations if several words made up a keyword. The complete search string in ISI Web of Science© was:

ts = (agricultur* OR farm* OR agroecosystem* OR cultivat* OR crop* OR horticultur* OR vegetab* OR orchard* OR “fruit tree*”)
AND ts = (“insect pest*” OR “pest insect*” OR “arthropod pest*” OR “damaging organism*” OR “damaging animal*” OR “damaging arthropod*” OR “beneficial organism*” OR “beneficial animal” OR “beneficial insect*” OR “natural enem*” OR “beneficial arthropod*” OR “pest parasit*” OR “pest predat*”)
AND ts = (hedgerow* OR “flower strip*” OR semi-natural OR “alley crop*” OR intercrop* OR tillage OR “crop rotation” OR “integrated pest management” OR IPM OR “crop diversi*” OR landscape* OR “low-intensity” OR “extensive” OR “small-scale” OR “large-scale” OR agroforest* OR organic OR low-input OR insecticid* OR low-dosage OR application timing OR “integrated fruit production” OR IFP OR “integrated production” OR refugia)
AND ts = (“biological control” OR cbc OR “pest control” OR “herbivory” OR “pest damage” OR “crop damage”)
*AND* **LANGUAGE:** (English) Indexes = SCI-EXPANDED, SSCI Timespan = 1990–2016
