# Supplementary material for: Investigating the (Mis)Match between Natural Pest Control Knowledge and the Intensity of Pesticide Use
Source: Insects. 2018 Jan 5;9(1):2. doi: 10.3390/insects9010002 (PMC5872267; doi:10.3390/insects9010002)
Supplement: Supplementary file 1 [file insects-09-00002-s001.zip › insects-229880 supplementay.zip/manuscript_supplementary_Mall et al/insects-229880_supplmentary-figures.docx]

Investigating the (mis)Match between Natural Pest Control Knowledge and the Intensity of Pesticide Use

David Mall, Ashley E. Larsen and Emily A. Martin


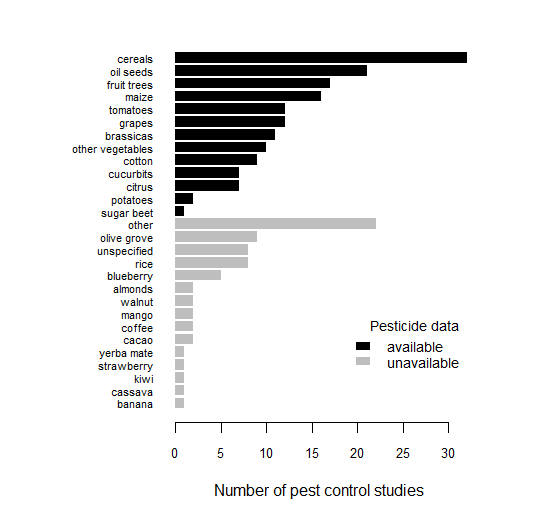


**Figure S1:** Total number of pest control-related studies per crop type. Crops for which pesticide use intensity data was unavailable (in grey) were not included in further analyses.


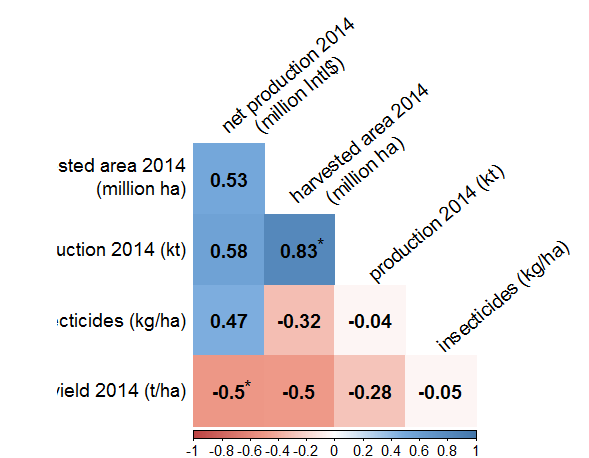
X

**Figure S2:** Pearson correlation matrix with all potential covariates. Stars (*) indicate significance (p<0.05). Red: negative correlation. Blue: positive correlation.


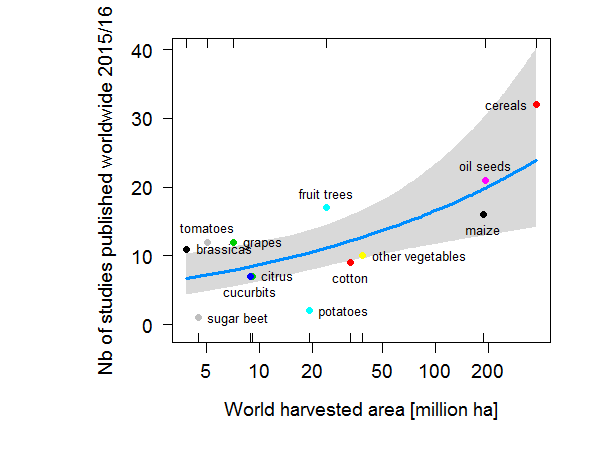


**Figure S3:** The number of studies on pest control detected 2015/16 as a function of total harvested area in 2014 in million ha worldwide. Note a log scale of world harvested area. Predicted values of the negative binomial generalized linear model are shown (Model 2, blue line; the 95% confidence interval is shown in gray).
